# Receipt of Recommended Follow-up Care After a Positive Lung Cancer Screening Examination

**DOI:** 10.1001/jamanetworkopen.2022.40403

**Published:** 2022-11-03

**Authors:** M. Patricia Rivera, Danielle D. Durham, Jason M. Long, Pasangi Perera, Lindsay Lane, Derek Lamb, Eman Metwally, Louise M. Henderson

**Affiliations:** 1 Division of Pulmonary and Critical Care Medicine, Department of Medicine, Rochester University Medical Center, Rochester, New York; 2 Wilmot Cancer Institute, University of Rochester, Rochester, New York; 3 Department of Radiology, The University of North Carolina, Chapel Hill; 4 Division of Cardiothoracic Surgery, Department of Surgery, University of North Carolina, Chapel Hill; 5 Lineberger Comprehensive Cancer Center, The University of North Carolina, Chapel Hill; 6 Department of Epidemiology, The University of North Carolina, Chapel Hill

## Abstract

**Importance:**

Maximizing benefits of lung cancer screening requires timely follow-up after a positive screening test. The American College of Radiology (ACR) Lung CT Screening Reporting and Data System (Lung-RADS) recommends testing and follow-up timing based on the screening result.

**Objective:**

To determine rates of and factors associated with recommended follow-up after a positive lung cancer screening examination by Lung-RADS category.

**Design, Setting, and Participants:**

This prospective cohort study of lung cancer screening examinations performed from January 1, 2015, through July 31, 2020, with follow-up through July 31, 2021, was conducted at 5 academic and community lung cancer screening sites in North Carolina. Participants included 685 adults with a positive screening examination, Lung-RADS categories 3, 4A, 4B, or 4X. Statistical analysis was performed from December 2020 to March 2022.

**Exposures:**

Individual age, race, sex, smoking exposure, year of lung cancer screening examination, chronic obstructive pulmonary disease, body mass index, referring clinician specialty, rural or urban residence.

**Main Outcomes and Measures:**

Adherence, defined as receipt of recommended follow-up test or procedure after the positive screen per ACR Lung-RADS timeframes: 6 months for Lung-RADS 3 and 3 months for Lung-RADS 4A. For Lung-RADS 4B or 4X, adherence was defined as follow-up care within 4 weeks, as ACR Lung-RADS does not specify a timeframe.

**Results:**

Among the 685 individuals included in this study who underwent lung cancer screening with low-dose computed tomography, 416 (60.7%) were aged at least 65 years, 123 (18.0%) were Black, 562 (82.0%) were White, and 352 (51.4%) were male. Overall adherence to recommended follow-up was 42.6% (292 of 685) and varied by Lung-RADS category: Lung-RADS 3 = 30.0% (109 of 363), Lung-RADS 4A = 49.5% (96 of 194), Lung-RADS 4B or 4X = 68.0% (87 of 128). Extending the follow-up time increased adherence: Lung-RADS 3 = 68.6% (249 of 363) within 9 months, Lung-RADS 4A = 77.3% (150 of 194) within 5 months, and Lung-RADS 4B or 4X = 80.5% (103 of 128) within 62 days. For Lung-RADS 3, recommended follow-up was less likely among those currently smoking vs those who quit (adjusted odds ratio [aOR], 0.48; 95% CI, 0.29-0.78). In Lung-RADS 4A, recommended follow-up was less likely in Black individuals vs White individuals (aOR, 0.35; 95% CI, 0.15-0.86). For Lung-RADS 4B or 4X, recommended follow-up was more likely in female individuals vs male individuals (aOR, 2.82; 95% CI, 1.09-7.28) and less likely in those currently smoking vs those who quit (aOR, 0.31; 95% CI, 0.12-0.80).

**Conclusions and Relevance:**

In this cohort study, adherence to recommended follow-up after a positive screening examination was low but improved among nodules with a higher suspicion of cancer and after extending the follow-up timeline. However, the association of extending the follow-up time of screen-detected nodules with outcomes at the population level, outside of a clinical trial, is unknown. These findings suggest that studies to understand why recommended follow-up is lower in Black individuals, male individuals, and individuals currently smoking are needed to develop strategies to improve adherence.

## Key Points

**Question:**

What are the rates and factors associated with adherence to recommended follow-up and adherence after an extended timeline following a positive lung cancer screening examination?

**Findings:**

In this cohort study of 685 adults with a positive lung cancer screening examination, overall adherence to recommended care was low but improved among higher-risk nodules and after extending the follow-up timeline. Adherence was statistically significantly lower in Black individuals, male individuals, and individuals currently smoking.

**Meaning:**

Although rates of follow-up were improved when the time was extended, the association between outcomes and delaying time to follow-up of screen-detected nodules is unknown.

## Teaser

This cohort study examines rates of and factors associated with recommended follow-up after a positive lung cancer screening examination by risk of cancer among adults.

## Introduction

The benefit in lung cancer mortality reduction achieved with low-dose computed tomography (LDCT) is due to detection of early-stage lung cancer, leading to improved 5-year survival.^1,2,3^ High adherence rates of 95% to 88% across multiple rounds of screening in the National Lung Screening Trial (NLST) and the Nederlands–Leuvens Longkanker Screenings Onderzoek (NELSON) trial^1,2^ contributed to the observed mortality reduction of screening. However, achieving the high adherence rates observed in clinical trials has been difficult at the population level, with reported rates varying from 28% to 82%.^4,5,6,7,8,9,10,11^ Many prior studies combined adherence to annual lung cancer screening (LCS) after a negative LDCT and adherence to recommended follow-up after a positive LDCT, leading to challenges in interpreting study results since recommended follow-up timing and imaging or diagnostic tests differ by screening result.^12^ Several studies reported that adherence to recommended follow-up is higher among individuals with a nodule detected on LDCT.^7,8^ Individuals found to have a nodule on LDCT have a higher likelihood of lung cancer; therefore, it is crucial to ensure appropriate follow-up occurs in this group.

The American College of Radiology (ACR) Lung CT Screening Reporting and Data System (Lung-RADS) lexicon provides recommendations for when individuals should return for follow-up with additional radiologic imaging or diagnostic procedures after a positive examination (Lung-RADS 3, 4A, 4B, or 4X category) (Table 1).^13^ However, while the ACR Lung-RADS provide a timeline for recommended follow-up of “probably benign” Lung-RADS 3 findings (CT within 6 months) and “suspicious” Lung-RADS 4A findings (LDCT or positron emission tomography/computed tomography [PET/CT] within 3 months), a current limitation of Lung-RADS is the lack of clear language on the timeline for obtaining additional diagnostic procedures including tissue sampling for “very suspicious” Lung-RADS 4B or 4X findings. In addition, the American Cancer Society National Lung Cancer Round Table (NLCRT) recently published proposed quality metrics for LCS programs that include compliance with follow-up recommendations per Lung-RADS categories defined as 6 months (±2 months) after a Lung-RADS 3 category and 3 months (±6 weeks) after a Lung-RADS 4 category. For Lung-RADS categories 4B and 4X, the evaluable metric was defined as the time in days from the finding on the LDCT to lung cancer diagnosis, although no specific timeline was provided (Table 1).^14^

**Table 1.** Lung-RADS Assessment Categories and Recommended Follow-up

ACR Lung-RADS Assessment^a^	Lung-RADS 3	Lung-RADS 4A	Lung-RADS 4B or 4X
Definition	Probably benign; risk of cancer 1%-2%	Suspicious; risk of cancer 5%-15%	Very suspicious; risk of cancer >15%
ACR-Lung-RADS recommended time to follow-up	6 mo	3 mo	Not specified
NLCRT Implementation Strategies Task Group^b^	6 mo ±2 mo	3 mo ±6 wk	Not specified
ACR recommended follow-up study/procedure^a^	LDCT	LDCT, PET/CT may be considered	Chest CT with/without contrast, PET/CT, and/or tissue sampling depending on probability of cancer

Abbreviations: ACR, American College of Radiology; CT, computed tomography; LDCT, low-dose computed tomography; Lung-RADS, Lung CT Screening Reporting and Data System; NLCRT, National Lung Cancer Round Table; PET/CT, positron emission tomography/computed tomography.

^a^ American College of Radiology: Lung CT Screening Reporting and Data System (Lung-RADS).^13^

^b^ Mazzone et al,^14^ 2021.

This study evaluated rates and factors associated with adherence to ACR Lung-RADS recommended follow-up and an extended timeline after a positive LDCT examination across 5 LCS sites. We focused on positive LCS examinations defined by Lung-RADS category to identify gaps in care and how interventions may need to be tailored among different groups based on demographic characteristics associated with decreased adherence.

## Methods

### Data Sources and Study Population

We collected data as part of the North Carolina Lung Screening Registry (NCLSR), an National Cancer Institute–funded registry that prospectively collects data on individuals undergoing LCS at participating locations throughout North Carolina (NC). Specifically, the NCLSR collects individual sociodemographic and risk factor information, screening examination and follow-up information, and outcomes data using abstracted electronic health records (EHRs) and radiology reports, data exports, and state cancer registry data. The NCLSR screening sites include academic and community sites, with patients screened through centralized and decentralized programs.

We examined the following LCS information: date of the LDCT examination (the LDCT that first identified a Lung-RADS 3 or 4 nodule leading to recommended further follow-up), first follow-up examination or procedure, and the Lung-RADS assessment category assigned by the interpreting radiologist. In addition, individuals’ sociodemographic information available at the time of the LDCT from the EHR was used and included: age (≤64 years, ≥65 years); race (Black, White); sex (female, male); smoking status (current, former, unknown); pack-years; if the individual formerly smoked, the time in years since quitting; presence of chronic obstructive pulmonary disease (COPD); weight and height used to calculate body mass index (BMI; categorized as underweight [<18.5], normal [18.5-24.9], overweight [25-29.9], and obese [≥30]); screening number categorized as baseline or subsequent screening examination; insurance status (private, Medicaid, Medicare, self-pay); and individuals’ residence classified as rural or urban using the individual’s zip code of residence and Rural-Urban Commuting Area (RUCA) classifications.^15^ Metropolitan designations were assigned as urban (RUCA = 1, 2, or 3) and nonmetropolitan designations were assigned as rural (RUCA = 4, 5, 6, 7, 8, 9, or 10). Clinician information was linked with the National Provider Index registry to determine clinician type (primary care practitioner, specialist, advanced nurse practitioner).^16^ These variables were included as we hypothesized that they might be associated with adherence to recommended follow-up. This study was approved by the University of North Carolina institutional review board with a waiver of informed consent because it was a secondary analysis of existing data. We followed the Strengthening the Reporting of Observational Studies in Epidemiology (STROBE) reporting guideline.

We included adults aged at least 18 years undergoing LDCT for LCS between January 1, 2015, and July 30, 2020, at 5 academic and community NCLSR sites with a positive LDCT defined as Lung-RADS category 3, 4A, 4B, or 4X. We required individuals to have at least 1 year of follow-up after their LDCT examination, during which follow-up examinations or procedures could be observed. Individuals with unknown race or race that was neither Black nor White were excluded from the analysis (n = 18) due to the small number of individuals in these groups.

### Measures

Lung-RADS assessment was collapsed to create 3 groups (Lung-RADS 3, Lung-RADS 4A, and a combination of Lung-RADS 4B and 4X). We combined the Lung-RADS 4B and 4X as they have the same recommended diagnostic follow-up per ACR Lung-RADS. We calculated the days from the LDCT examination until the occurrence of the recommended follow-up examination or procedure. We used ACR Lung-RADS recommendations to define follow-up of Lung-RADS 3 and Lung-RADS 4A findings (Table 1).^13^ For Lung-RADS 4B or 4X, the ACR Lung-RADS recommends chest CT with or without contrast, PET/CT, and/or tissue sampling but does not provide a follow-up time, only stating “immediately.” Therefore, we selected 4 weeks as the recommended time to follow up for Lung-RADS 4B and 4X findings (Table 1). In addition, we created a second set of follow-up time intervals to extend the recommended follow-up timeframes (referred to as extended time intervals). We defined the extended time intervals as within 6 to 9 months (for Lung-RADS 3) and 3 to 5 months (for Lung-RADS 4A) of the LDCT examination. For Lung-RADS 4B and 4X, we used 62 days, based on recommendations from the British Thoracic Society (BTS) guidelines for “time from abnormal radiograph to surgery” for non–screen detected radiographic findings suspicious for lung cancer.^17^ Lastly, we evaluated follow-up outside the extended period looking up to 1 year after the LDCT screening examination (Table 1).

### Statistical Analysis

We described the characteristics of individuals by Lung-RADS category. We compared the frequency of return for follow-up within the recommended timeframe for the Lung-RADS category and by age, race, and sex. We tested for differences in median follow-up times using the Wilcoxon test. We described the timing of return based on the recommended follow-up times and extended time intervals for each Lung-RADS category. Specifically, we described the proportions of patients within each Lung-RADS category who returned: within the recommended timeframe, within the extended time interval, outside the extended time interval, and who were not observed to have returned for follow-up. We used multivariate logistic regression to examine factors associated with receipt of follow-up within the ACR Lung-RADS recommended intervals after a positive LCS examination separately for the 3 Lung-RADS groups and reported adjusted odds ratios (OR) and 95% CIs. The adjusted models included age, race, sex, smoking status, preexisting COPD, BMI, residence type (rural vs urban), LCS examination year, LCS imaging site, and referring clinician type (primary care physician [PCP], advanced practice [APP], vs specialist). The significance threshold was α = .05 and all tests were 2-sided. Analyses were conducted using SAS version 9.4 (SAS Institute) from December 2020 to March 2022.

## Results

Among the 685 individuals included who underwent LCS with LDCT and were found to have a positive result, 416 (60.7%) were aged at least 65 years, 123 (18.0%) were Black, 562 (82.0%) were White, and 352 (51.4%) were male (Table 2). Lung-RADS 3 category nodules were reported for 363 individuals, Lung-RADS 4A for 194 individuals, and Lung-RADS 4B or 4X for 128 individuals. The distribution of patient characteristics varied according to the Lung-RADS category. For example, younger individuals (aged less than 65 years) tended to have Lung-RADS 3 nodules (61.0% [164 of 269]), whereas older individuals (aged greater than 65 years) tended to have Lung-RADS 4A, 4B or 4X nodules (52.2% [217 of 416]). Black individuals tended to have higher Lung-RADS categories than White individuals (22.0% vs. 18.0% with Lung-RADS 4B or 4X). Individuals who were currently smoking were more likely to have Lung-RADS 3 nodules compared with those who formerly smoked (56.2% vs. 48.4%).

**Table 2.** Characteristics of Individuals With Positive Lung Cancer Screening Examinations by Lung-RADS Category, North Carolina Lung Screening Registry 2015 to 2020

Characteristic	All patients, No. (column %) (N = 685)	Patients, No. (row %)		
		Lung-RADS 3 (n = 363)	Lung-RADS 4A (n = 194)	Lung-RADS 4B or 4X (n = 128)
Age group, y				
<65	269 (39.3)	164 (61.0)	74 (27.5)	31 (11.5)
≥65	416 (60.7)	199 (47.8)	120 (28.8)	97 (23.3)
Race				
Black	123 (18.0)	59 (48.0)	37 (30.1)	27 (22.0)
White	562 (82.0)	304 (54.1)	157 (27.9)	101 (18.0)
Sex				
Female	333 (48.6)	176 (52.9)	96 (28.8)	61 (18.3)
Male	352 (51.4)	187 (53.1)	98 (27.8)	67 (19.0)
Smoking status				
Current	347 (51.6)	195 (56.2)	88 (25.4)	64 (18.4)
Former	326 (48.4)	158 (48.4)	104 (31.9)	64 (19.6)
Missing^a^	12	10	2	0
Smoking pack-years, median (IQR) [range]	40 (28-54) [1.8-318]	40 (27-52) [2-129]	43 (30-56) [1.8-318]	45 (30-60) [5-110]
Time since quit, median (IQR) [range], y	5 (2-10) [0-37]	5 (2-11) [0-36]	5 (2-10) [0-29]	3.5 (2-9) [0-37]
Year of LDCT screening examination				
2015-2017	259 (37.8)	133 (36.6)	71 (36.6)	55 (43.0)
2018-2020	426 (62.2)	230 (63.4)	123 (63.4)	73 (57.0)
COPD				
No	282 (41.2)	149 (52.8)	80 (28.4)	53 (18.8)
Yes	386 (57.8)	200 (51.8)	111 (28.8)	75 (19.4)
Missing^a^	17	14	3	0
BMI				
<25	229 (34.7)	110 (48.0)	75 (32.8)	44 (19.2)
≥25	430 (65.3)	239 (55.6)	113 (26.3)	78 (18.1)
Missing^a^	26	14	6	6
Screening No.				
Baseline	529 (77.2)	296 (55.9)	139 (26.4)	94 (17.8)
Subsequent	156 (22.8)	67 (42.9)	55 (35.3)	34 (21.8)
Individual insurance				
Private	165 (24.1)	100 (60.6)	45 (27.3)	20 (12.1)
Medicaid	21 (3.1)	11 (52.4)	6 (28.6)	4 (19.0)
Medicare	470 (68.6)	236 (50.2)	136 (28.9)	98 (20.9)
Self-pay	29 (4.2)	16 (55.2)	7 (24.1)	6 (20.7)
Individual residence				
Urban	345 (50.4)	182 (52.8)	111 (32.2)	52 (15.0)
Rural	340 (49.6)	181 (53.2)	83 (24.4)	76 (22.4)
Referring clinician specialty				
Primary care practitioner	259 (37.8)	128 (49.4)	76 (29.2)	55 (21.2)
Specialist	136 (19.9)	81 (59.6)	38 (27.9)	17 (12.5)
Advanced practice provider	274 (40.0)	146 (53.3)	78 (28.5)	50 (18.2)
Missing^a^	16	8	2	6

Abbreviations: BMI, body mass index; COPD, chronic obstructive pulmonary disease; LDCT, low-dose computed tomography; Lung-RADS, Lung CT Screening Reporting and Data System.

^a^ Missing counts excluded from percentages.

### Adherence to Recommended Follow-up Care After a Positive LDCT

Overall adherence to recommended follow-up per the ACR Lung-RADS recommended timeline for Lung-RADS category 3 and 4A nodules, and the 4-week timeline we selected for Lung-RADS 4B or 4X, was 42.6% (292 of 685), ranging from 30.0% (109 of 363) for Lung-RADS 3, 49.5% (96 of 194) for Lung-RADS 4A, and 68.0% (87 of 128) for Lung-RADS 4B or 4X (Table 3). Extended time intervals increased overall adherence to follow-up to 73.3% (502 of 685), ranging from 68.6% (249 of 363) for Lung-RADS 3, 77.3% (150 of 194) for Lung-RADS 4A, and 80.5% (103 of 128) for Lung-RADS 4B or 4X. Extending follow-up to 1 year after the LDCT screening examination further improved the overall adherence rate to 82.3% (654 of 685), ranging from 75.8% (275 of 363) for Lung-RADS 3, 87.6% (170 of 194) for Lung-RADS 4A, and 93.0% (119 of 128) for Lung-RADS 4B or 4X.

**Table 3.** Receipt of Recommended Follow-up by Lung-RADS Category and Extended Time Intervals

Lung-RADS categories with recommended follow-up tests/procedures and timing	Patients, No. (%)
**All**	
No.	685
Overall adherence to ACR recommended follow-up time (Lung-RADS 3 and 4A) and 4 wk for Lung-RADS 4B or 4X	292 (42.6)
Overall adherence to extended period for Lung-RADS 3, 4A, 4B, or 4X	502 (73.3)
Overall adherence to follow-up after extended period to 1 y for Lung-RADS 3, 4A, 4B, or 4X	564 (82.3)
**Lung-RADS 3: Probably benign**	
No.	363
Recommended follow-up: LDCT within 6 mo^a^	109 (30.0)
Follow-up in extended period: LDCT in 6-9 mo	140 (38.6)
Follow-up outside extended period: LDCT>9 mo to 1 y	26 (7.2)
No observed LCS follow-up within 1 y	88 (24.2)
Time until follow-up, median (IQR), d	
Among those with LDCT within 6 mo	161 (112-176)
Among those with LDCT within 1 y	199 (189-243)
**Lung-RADS 4A: Suspicious**	
No.	194
Recommended Follow-up: LDCT or PET/CT within 3 mo^a^	96 (49.5)
Follow-up in extended period: LDCT or PET/CT within 3-5 mo	54 (27.8)
Follow-up outside extended period: LDCT or PET/CT>5 mo to 1 y	20 (10.3)
No observed LCS follow-up within 1 y	24 (12.4)
Time until follow-up, median (IQR), d	
Among those with LDCT or PET/CT within 3 mo	30.5 (14.5-72.5)
Among those with LDCT or PET/CT within 1 y	101 (92-133)
**Lung-RADS 4B or 4X: Very suspicious**	
No.	128
Recommended follow-up: Chest CT with or without contrast or PET/CT or biopsy within 4 wk^b^	87 (68.0)
Follow-up in extended period: Chest CT with or without contrast or PET/CT or biopsy (62 d)^c^	16 (12.5)
Follow-up outside extended period: Chest CT with or without contrast or PET/CT or biopsy >62 d to 1 y	16 (12.5)
No observed LCS follow-up within 1 y	9 (7.0)
Time until follow-up, median (IQR), d	
Among those with chest CT with or without contrast or PET/CT or biopsy within 4 wk	14 (10-20)
Among those with chest CT with or without contrast or PET/CT or biopsy within 1 y	60.5 (41.5-116.5)

Abbreviations: ACR, American College of Radiology; CT, computed tomography; LCS, lung cancer screening; LDCT, low-dose computed tomography; Lung-RADS, Lung CT Screening Reporting and Data System; PET, positron emission tomography.

^a^ Per American College of Radiology Lung-RADS.

^b^ Timeline we selected for Lung-RADS 4B or 4X as ACR Lung-RADS does not give a specific timeframe.

^c^ Per British Thoracic Society recommended timeline from “abnormal radiograph to surgery” for non–screen detected radiographic findings suspicious for lung cancer.

### Factors Associated With Recommended Follow-up

Among individuals with a Lung-RADS 3 result, those currently smoking were less likely to obtain follow-up within the ACR recommended timeline than those who formerly smoked (aOR, 0.48; 95% CI, 0.29-0.78) (Table 4 and Table 5). In those with a Lung-RADS 4A result, Black individuals were 65% less likely to receive ACR-recommended follow-up vs White individuals (aOR, 0.35; 95% CI, 0.15-0.86) (Table 4 and Table 5). Among individuals with a Lung-RADS 4B or 4X result, female individuals were approximately 3 times more likely than male individuals to receive recommended follow-up (aOR, 2.82; 95% CI, 1.09-7.28) (Table 4 and Table 5). In addition, those who currently smoked were less likely to receive recommended follow-up than those who formerly smoked (aOR, 0.31; 95% CI, 0.12-0.80) (Table 4 and Table 5). Insurance status was not associated with receipt of recommended follow-up in univariate analyses and was not included in the adjusted models.

**Table 4.** Proportion of Individuals who Received Follow-up by Smoking Status, Race, and Sex, According to Lung-RADS Category

	Patients, No. (Col %)					
	Lung-RADS 3		Lung-RADS 4A		Lung-RADS 4B/4X	
**Age group**	<65 y	≥65 y	<65 y	≥65 y	<65 y	≥65 y
Follow-up						
Within recommended period	50 (30.5)	59 (29.7)	29 (39.2)	67 (55.8)	19 (61.3)	68 (70.1)
Within extended period	58 (35.4)	82 (41.2)	20 (27.0)	34 (28.3)	4 (12.9)	12 (12.4)
Within 1-y	18 (11.0)	8 (4.0)	11 (14.9)	9 (7.5)	3 (9.7)	13 (13.4)
No observed LCS follow-up within 1 y	38 (23.2)	50 (25.1)	14 (19.0)	10 (8.4)	5 (16.2)	4 (4.0)
**Race**	**Black**	**White**	**Black**	**White**	**Black**	**White**
Follow-up						
Within recommended period	15 (25.4)	94 (30.9)	11 (29.7)	85 (54.1)	19 (70.4)	68 (67.3)
Within extended period	25 (42.4)	115 (37.8)	15 (40.5)	39 (24.8)	1 (3.7)	15 (14.9)
Within 1-y	4 (6.8)	22 (7.2)	4 (10.8)	16 (10.2)	5 (18.5)	11 (10.9)
No observed LCS follow-up within 1 y	15 (25.4)	73 (24.0)	7 (18.9)	17 (10.9)	2 (7.4)	7 (7.0)
**Sex**	**Female**	**Male**	**Female**	**Male**	**Female**	**Male**
Follow-up						
Within recommended period	56 (31.8)	53 (28.3)	47 (49.0)	49 (50.0)	46 (75.4)	41 (61.2)
Within extended period	68 (38.6)	72 (38.5)	27 (28.1)	27 (27.6)	7 (11.5)	9 (13.4)
Within 1-y	14 (8.0)	12 (6.4)	12 (12.5)	8 (8.2)	5 (8.2)	11 (16.4)
No observed LCS follow-up within 1 y	38 (21.6)	50 (26.7)	10 (10.4)	14 (14.3)	3 (4.9)	6 (9.0)
**Smoking status**	**Current**	**Former**	**Current**	**Former**	**Current**	**Former**
Follow-up						
Within recommended period	46 (23.6)	61 (38.6)	38 (43.2)	57 (54.8)	39 (60.9)	48 (75.0)
Within extended period	74 (38.0)	62 (39.2)	22 (25.0)	32 (30.8)	9 (14.0)	7 (10.9)
Within 1-y	17 (8.7)	9 (5.7)	13 (14.8)	7 (6.7)	10 (15.6)	6 (9.4)
No observed LCS follow-up within 1 y	58 (29.7)	26 (16.5)	15 (17.0)	8 (7.7)	6 (9.4)	3 (4.7)

Abbreviations: LCS, lung cancer screening; Lung-RADS, Lung CT Screening Reporting and Data System.

**Table 5.** Factors Associated With Receipt of Recommended Follow-up After a Positive Lung Cancer Screening Examination by Lung-RADS Category

Characteristic	OR (95% CI)^a^		
	Lung-RADS 3	Lung-RADS 4A	Lung-RADS 4B or 4X
Age, y			
<65	1.17 (0.72-1.90)	0.66 (0.35-1.27)	1.10 (0.36-3.32)
≥65	1 [Reference]	1 [Reference]	1 [Reference]
Race			
Black	0.77 (0.39-1.54)	0.35 (0.15-0.86)	1.45 (0.48-4.41)
White	1 [Reference]	1 [Reference]	1 [Reference]
Sex			
Female	1.10 (0.68-1.79)	1.08 (0.58-2.00)	2.82 (1.09-7.28)
Male	1 [Reference]	1 [Reference]	1 [Reference]
Smoking status			
Current	0.48 (0.29-0.78)	0.73 (0.39-1.37)	0.31 (0.12-0.80)
Former	1 [Reference]	1 [Reference]	1 [Reference]
Year of examination			
2015-2017	0.75 (0.45-1.25)	1.03 (0.54-1.99)	0.91 (0.33-2.46)
2018-2020	1 [Reference]	1 [Reference]	1 [Reference]
COPD			
Yes	1.37 (0.84-2.25)	1.80 (0.96-3.37)	2.65 (0.95-7.42)
No	1 [Reference]	1 [Reference]	1 [Reference]
BMI			
<25	1 [Reference]	1 [Reference]	1 [Reference]
≥25	1.07 (0.63-1.81)	1.45 (0.77-2.73)	0.78 (0.29-2.14)
Referring clinician			
PCP or APP	0.62 (0.34-1.10)	0.82 (0.37-1.83)	2.15 (0.58-7.94)
Specialist	1 [Reference]	1 [Reference]	1 [Reference]
Individual residence			
Urban	1 [Reference]	1 [Reference]	1 [Reference]
Rural	0.87 (0.51-1.50)	0.78 (0.38-1.61)	2.21 (0.67-7.25)

Abbreviations: APP, advanced practice provider; BMI, body mass index; COPD, chronic obstructive pulmonary disease; Lung-RADS, Lung CT Screening Reporting and Data System; OR, odds ratio; PCP, primary care physician.

^a^ Adjusted for all characteristics in the table and lung cancer screening imaging site.

## Discussion

This multicenter cohort study of individuals undergoing LCS examined factors associated with adherence to the ACR Lung-RADS recommended follow-up care, after an extended timeline, and up to 1 year after the LDCT screening examination. Our study found that overall adherence to recommended follow-up after a positive LDCT was low but improved with increasing Lung-RADS category. Furthermore, we found differences in rates of recommended adherence based on race, sex, and smoking status. Our results highlight gaps in recommended follow-up according to level of suspicion for lung cancer per Lung-RADS and suggest an area of future research focused on the consequences of decreased adherence to recommended follow-up on lung cancer mortality at the population level.^18^

Adherence to positive LCS examinations at the population level has been heterogeneous.^4,5,6,7,8,9,10,11^ In a recent meta-analysis, the pooled adherence rate in 557 individuals with Lung-RADS 3 or 4 was 74% (95% CI, 65%-83%). However, this analysis did not stratify into Lung-RADS 4A vs 4B or 4X, and the suspicion for cancer varies in these groups.^19^ Factors associated with increased adherence rates after LCS examinations include higher Lung-RADS assessment after a baseline LDCT,^20,21^ White race,^6,11^ type of program (centralized vs decentralized or having a nurse navigator),^10,22,23^ and former smoking status.^20^ Two studies reported individuals with Lung-RADS 3 and Lung-RADS 4 were more likely to be adherent to recommended follow-up than those with Lung-RADS 1, with Lung-RADS 3 individuals 3 to 5 more likely and Lung-RADS 4 individuals 14 to 29 times more likely.^20,21^ Our study had similar findings with higher adherence rates in individuals with Lung-RADS 4 vs Lung-RADS 3 category nodules. Furthermore, we found adherence rates were higher among female individuals with Lung-RADS 4B, White individuals with Lung-RADS 4A category nodules, and those who formerly smoked with Lung-RADS 3 and 4B or 4X nodules.

A different meta-analysis exploring racial differences in LCS adherence reported lower adherence rate to follow-up in Black vs White individuals across all Lung-RADS groups, with an OR of 0.56 (95% CI, 0.45-0.70) in the Lung-RADS 3 or 4 groups.^6^ In a more recent retrospective study of 6134 individuals undergoing LCS, of whom 992 (16.2%) had a positive baseline screening examination, follow-up to recommended care after a positive LDCT was lower at decentralized programs compared with centralized programs (63.9% vs 74.6%; *P* < .001), however, no difference in follow-up by race was observed.^23^

Differences in how studies define adherence (ACR Lung-RADS recommended follow-up time vs extended time interval vs any follow-up during an observed time interval) to recommended follow-up either with repeat imaging or biopsy after the screening LDCT has led to substantial variability in the reporting of adherence rates.^19^ In a recent study by Kim et al,^23^ the overall adherence rate for recommended follow-up after a positive LDCT was 69.9%. The authors used a follow-up timeline from the index LDCT of 4 to 9 months for Lung-RADS 3, 1 to 5 months for Lung-RADS 4A, and within 5 months for Lung-RADS 4B or 4X.^23^ A Veterans Hospital Administration (VHA) health care system study reported adherence following the ACR Lung-RADS recommendations and also evaluated adherence rates using an extended timeline following recommendations from an expert consensus on accepted delays in evaluation of screen-detected nodules during the COVID-19 pandemic,^24^ defined as: (1) within 2 months before or 3 months after the index LDCT for Lung-RADS 3; (2) within 1 to 5 months after the index LDCT for Lung-RADS 4, and (3) within 0 to 5 months after the index LDCT for Lung-RADS 4B or 4X.^11^ Overall adherence to ACR-recommended follow-up after a positive LDCT was 63.1%, and an additional 13.1% of individuals returned for follow-up during the extended timeline. Individuals with high-risk findings (Lung-RADS 4) were less likely to have delayed or no evaluation (OR, 0.35; 95% CI, 0.28-0.43 for Lung-RADS 4 vs Lung-RADS 1).^11^ Similarly, we evaluated adherence to the ACR Lung-RADS recommended follow-up time for Lung-RADS 3 and 4A nodules, and to a 4-week follow-up timeline we selected for high-risk nodules (Lung-RADS 4B or 4X) and to follow-up within an extended period and 1 year after the LDCT screening examination. Using the extended time interval resulted in higher adherence rates for Lung-RADS 3, Lung-RADS 4A, and Lung-RADS 4B or 4X. In addition, extending follow-up to 1 year after the positive LDCT increased the overall adherence rate to 82.3%.

Although an extended, more liberal timeline for follow-up of a positive LDCT improves adherence rates, the impact of delays in follow-up care of screen-detected nodules is not well-known. It is likely that extending the follow-up timeline of Lung-RADS 3 nodules, very low risk of cancer, will not affect outcomes substantially. However, there is not enough evidence about the implications of extending the follow-up timeline for Lung-RADS 4 nodules, particularly high-risk Lung-RADS 4B or 4X nodules where the risk of cancer is greater than 15%.^13^ One challenge is that the recommended optimal time from lung cancer diagnosis to treatment is quite variable.^17,25,26^ An association between worse survival and longer interval between the diagnosis of early-stage non–small cell lung cancer (NSCLC) and surgery has been reported.^27,28,29^ A retrospective analysis of 2 cohorts from the NLST (n = 452) and the National Cancer Data Base (NCDB; n = 80 086) evaluated the association between outcomes and early (0 to 30 days) vs delayed (90 to 120 days) surgery for stage I adenocarcinoma and squamous cell cancer (SCC).^27^ No significant difference was found in survival for stage 1A1 adenocarcinoma and 1A1-1A3 SCC (all *P* > .13); however, for larger tumors, stage 1A2-1B adenocarcinoma and stage 1B SCC, delayed surgery was associated with worse survival (all *P* < .004).^27^ In a retrospective study of 4984 patients with stage 1A SCC using the NCDB from 2006 to 2011, surgery 38 days or more after a diagnosis of SCC was associated with worse 5-year survival than surgery within 30 days of diagnosis (hazard ratio [HR], 1.13; 95% CI, 1.02-1.25; *P* = .02).^28^ Another retrospective cohort study using data from the VHA system from 2006 to 2016 evaluated factors associated with worse survival in 9904 patients with stage I NSCLC. Mean time to surgery (TTS), defined as time from the abnormal chest CT to surgery, was 38.6 days. Longer TTS, greater than 12 weeks, was associated with worse survival; HR for recurrence increased by 0.4% (HR, 1.004; 95% CI, 1.001-1.006; *P* = .002) for each week of delay after 12 weeks.^29^

This study provides data on rates of ACR-recommended follow-up after a positive LDCT. In addition, we provide data on follow-up rates after an extended timeline and up to 1 year after the LDCT screening examination. Our results align with previous studies showing overall adherence to recommended follow-up after a positive LDCT is low but improves with increasing Lung-RADS category. Furthermore, increasing the time to follow-up outside what is recommended by the ACR Lung-RADS criteria improved the rate of recommended follow-up. We also found disparities in follow-up care among Black individuals with Lung-RADS 4A nodules, decreased adherence in men with Lung-RADS 4B or 4X nodules, and decreased adherence in individuals currently smoking with Lung-RADS 3 and 4B or 4X nodules.

Strategies to improve follow-up after a positive screening examination should focus on improving adherence to ACR-recommended follow-up for Lung-RADS 3 and 4A nodules with special attention on Black individuals, men, and those currently smoking to obtain timely follow-up. In addition, because there is no clear recommendation for the appropriate time to follow-up of Lung-RADS 4B and 4X nodules, consensus is needed on the appropriate follow-up timeline for these high-risk nodules. This is crucial given data from other studies suggesting delays in early-stage lung cancer care are associated with worse survival.

### Strengths and Limitations

Our study has several strengths. First, we included data from 5 LCS imaging sites, academic and community-based practices, to expand the generalizability of our findings. Second, we stratified results by Lung-RADS category rather than combining all positive results, which allowed us to evaluate more homogeneous groups. Third, we used a more stringent timeline of 4 weeks for recommended follow-up and 62 days for extended follow-up of high-risk, Lung-RADS 4B or 4X nodules and showed most individuals returned for recommended follow-up.

This study also has some limitations, which include the study being conducted in 1 geographic area, and there may be differences in patterns of care across the US. In addition, individuals may have received follow-up care outside the location where they were screened, and this follow-up would not be captured in our data.

## Conclusions

This cohort study found that follow-up to ACR recommendations for Lung-RADS 3 and 4A nodules and 4 weeks for Lung-RADS 4B or 4X nodules is low but improved among nodules with a higher suspicion for lung cancer and extended follow-up timelines. The association between outcomes and extending timelines is unknown, yet data from other studies suggest the potential for worse outcomes due to delays in surgery for some early-stage NSCLCs. Therefore, efforts should be made to better define the recommended timeline for follow-up of high-risk screen-detected nodules, Lung-RADs 4B and 4X, and to understand why recommended follow-up after a positive result are lower in Black individuals, male individuals, and those who currently smoke.
